# Vitamin D and Calcium Supplementation in Elderly Patients Suffering Fragility Fractures; The Road not Taken

**DOI:** 10.2174/1874325001711011230

**Published:** 2017-10-31

**Authors:** Aaron K. Saini, Edward J.C. Dawe, Simon M. Thompson, John W. Rosson

**Affiliations:** 1 Specialist Registrar in Trauma and Orthopaedics, Royal Surrey County Hospital, Guildford, Surrey, UK; 2 Specialist Registrar in Trauma and Orthopaedics, Royal Surrey County Hospital, Guildford, Surrey, UK, Honorary Clinical Lecturer, Brighton and Sussex Medical Schools; 3 Locum Consultant Orthopaedic Surgeon, Chelsea and Westminster Hospital, London, UK; 4 Consultant Orthopaedic Surgeon, Royal Surrey County Hospital, Guildford, Surrey, UK

**Keywords:** Fragility fracture, Hip fracture, Vitamin D supplementation

## Abstract

**Background:**

Calcium and Vitamin D supplementation in elderly patients may decrease the risk of hip fracture by up to one-third. Many patients suffering fragility fractures do not go on to receive this treatment despite clear recommendations from the National Institute for Health and Clinical Excellence (NICE). The aim of this study was to audit the proportion of patients admitted with a hip fracture who had suffered a previous fragility fracture and were taking calcium and vitamin D supplements, with the standard being that all of these patients should have been taking bone protection. We also aimed to assess the Vitamin D levels of patients admitted with a hip fracture to our unit.

**Methods:**

Patients were prospectively added to a database over a 12-month period. Serum vitamin D levels (25-OH D3) were measured on admission and case-notes were reviewed for pre-injury social function and mobility.

**Results:**

147 patients were included in the study. Median age was 85 years (Interquartile range 79 – 90 (Range 53 – 100 years)). Only eighteen patients (11.4%) were taking calcium and vitamin D supplementation on admission. Forty seven patients (29%) had documented evidence of a previous fragility fracture within the last seven years. Only fourteen of these patients (19%) were receiving calcium and vitamin D supplementation. One hundred and twenty two patients were deficient in Vitamin D (76%). Twenty five patients (16%) had insufficient Vitamin D. Only the remaining 14 patients (8%) had sufficient vitamin D.

**Conclusion:**

Vitamin D deficiency is endemic amongst patients suffering hip fractures. Very few patients who had suffered a previous fragility fracture were taking Calcium and Vitamin D supplements when admitted with a hip fracture several years later. This is an opportunity missed.

## 1 INTRODUCTION

There is increasingly clear evidence for the benefits of vitamin D and calcium supplementation in patients suffering fragility fractures. These have been recommended by the National Institute for Health and Clinical Excellence (NICE) [1]. The presence of a fragility fracture often heralds the presence of another more significant injury such as a hip fracture in the coming years [2]. Hence, any clinician treating a patient who suffers a fragility fracture is presented with an opportunity to reduce that individual’s likelihood of subsequent fracture. This opportunity is however commonly missed. A review by Giangregorio *et al.* [3] found that only 5-30% of patients had a diagnosis of osteoporosis prior to an index fragility fracture, highlighting the importance of this event as a diagnostic and intervention opportunity. The primary aim of this study was to measure the proportion of patients admitted with a hip fracture that had suffered a previous fragility fracture but had not been started on Calcium and Vitamin D supplements. The secondary aim of this study was to identify the incidence of Vitamin D insufficiency and deficiency in this group of patients.

## 2 METHODS

Patients presenting to a single centre with a fracture of the femoral neck were prospectively added to a database for twelve months between February 2011 and February 2012. Case-notes were reviewed to assess pre-admission medications and usual level of independence.

The null hypothesis was that there would be no difference in the proportion of patients receiving Calcium and Vitamin D supplements on admission between those who suffered a previous fragility fracture and those who did not.

Serum levels of 25-hydroxycholecalciferol [25(OH)D_3_] are routinely measured on admission blood samples. There is no consensus on precise values for Vitamin D insufficiency or deficiency but for the purposes of this study the thresholds recently stated by Pearce *et al.* are used. Greater than 75nmol/l of 25(OH)D_3_ was considered optimal. A level between 50nmol/l and 75nmol/l was considered sufficient. Insufficiency was defined as a level between 25nmol/l and 50 nmol/l with deficiency defined as a level below 25nmol/l [4].

Radiographs were reviewed to assess for evidence of other insufficiency fractures in the preceding seven years. Specific injuries included were fractures of the femoral neck, pelvis, distal radius and proximal humerus. Vertebral wedge compression fractures demonstrated on spinal radiographs were also included.

All patients in this study received joint care with an Orthogeriatrician and were discharged on calcium and vitamin D supplementation along with further bone protection where appropriate.

Categorical variables were compared using a Chi squared test or Fisher exact test where appropriate. Statistical analysis was carried out using the Medcalc software package (V11.2.1.0).

## 3 RESULTS

One hundred and sixty one patients were included in the study. Median age was 85 years (Interquartile range 79–89 (Range 53–100 years)). Demographics are shown in Table (**1**).

Median vitamin D level was 32nmol/l (Inter-quartile range 18-50nmol/l (Range 10-310nmol/l)). Within this sample, 63 patients had Vitamin D deficiency (39%) whilst a further 59 patients (36.6%) had insufficient Vitamin D. Twenty five patients (15.5%) had a vitamin D level within the acceptable range and only 14 patients (8.7%) had optimal levels. These results are illustrated in Table (**2**).

Only eighteen patients (11.4%) were taking calcium and vitamin D supplementation on admission. Fourteen patients (8%) were taking other bone protection (all taking alendronic acid except one patient taking risedronate, (Table **3**).

Forty seven patients (29%) had documented evidence of a previous fragility fracture within the last seven years, with the distribution of location summarised in Table (**4**). Only nine of these patients (19%) were receiving calcium and vitamin D supplementation, (Table **5**).

There was no significant difference in the proportion of patients receiving Calcium and Vitamin D supplementation on admission between patients who had suffered a previous fragility fracture and those who had not (P=0.074).

## 4 DISCUSSION

The incidence of hip fracture in the United Kingdom is increasing and is estimated to reach 100,000 fractures a year by 2033 [5]. Low vitamin D concentrations have been reported in the majority of patients suffering hip fractures in northern European countries [6-9]. This is reflected in our results as 76 percent of patients possessed either Vitamin D insufficiency or deficiency. Vitamin D supplementation in elderly patients has been the subject of a number of large scale studies assessing the efficacy of this intervention in certain high-risk groups [10-14]. The benefits in terms of fracture prevention are twofold; elderly patients receiving high-dose Vitamin D and calcium supplements not only develop improvements in bone density but also develop improved balance and are less likely to suffer falls [15]. The risk of subsequent hip fracture may hence reduce by up to 30%. The results of this study show that approximately one third of patients had undergone a radiological investigation within the last seven years which demonstrated an insufficiency fracture. However, only nineteen percent were then commenced on calcium and vitamin D supplements. Our data did not show a statistically significant difference between the proportions of patients receiving calcium and vitamin D supplements between those who had sustained a previous fragility fracture and those who have not. However, this does not prove a lack of association especially in the context of the sample size. This study highlights the need for a change in practice at national level in order to ensure all patients suffering fragility fractures are treated appropriately. A recent study suggests that the probability of being prescribed anti-osteoporosis medication *after* sustaining a hip fracture is improving [16]. The group found that in the U.K. in 2000 7% of these patients received bisphosphonates with Vitamin D and calcium supplements. While this improved to 46% in 2010, it is still far below the desired level.

### 4.1 Evidence For the Benefit of Supplementation

There is a wealth of published literature on the potential benefits of supplementation in patients with Vitamin D insufficiency or deficiency. This includes clear evidence for reductions in mortality through effects on cardiovascular disease and hypertension as well as a reduction in the risk of colorectal cancer [10]. There is weaker evidence for benefits in numerous conditions such as osteoarthritis, insulin resistance and other types of cancers [10]. In the doses required to prevent subsequent hip fracture, the benefits of vitamin D and calcium supplementation are clearly in excess of the risks associated with their usage.

Several randomised trials have looked at the effect of vitamin D and Calcium supplementation on preventing fracture in elderly populations. Further episodes of significant injuries such as hip fracture have been best prevented in those studies where participants were receiving greater than 800IU/day of Vitamin D3 along with supplemental calcium [15]. Chapuy *et al.* randomised 3270 elderly institutionalised women to receive either 800IU/day of Vitamin D with 1.2g elemental Calcium or placebo [14]. The rate of hip fracture was 43% lower in the supplementation group than the placebo group. Furthermore, bone densitometry of the femoral neck showed an increase of 2.7% in the supplemented group compared to decreased density of 4.7% in the placebo group. The same authors later conducted a cost-effectiveness study on their results and found a cost saving of €79000 per thousand patients [17]. This effect size is however significantly dependent upon the group of patients studied and the level of supplementation provided.

### 4.2 Risks of Calcium and Vitamin D Supplementation

There are a number of side effects which occur in patients taking calcium and vitamin D supplements. These include epigastric discomfort, sweating, constipation and hypercalciuria although overall rates of these are low, occurring in less than 5% of patients [18]. Only one randomised controlled trial has so far detected an increase in the rate of nephrolithiasis in patients receiving Calcium and Vitamin D supplements. This was the Women Health Initiative trial involving over 36,000 patients; by far the largest available study. It is likely that if this effect truly exists that there is a very small effect size and this trial has been the only one able to detect it [10]. There is no clear consensus on whether vitamin D taken alone produces the same effect in reducing fracture risk as it does when taken with supplementary calcium. Some studies have however found a modest increase in the risk of suffering cardiovascular events when taking calcium supplementation, in particular myocardial infarction [19].

### 4.3 Who Should Take Responsibility?

Consideration of overall bone health should form part of the assessment of any patient suffering a fragility fracture. Opportunities to shoulder this responsibility are available to medical staff assessing patients with a fracture in the accident and emergency department, orthopaedic staff in the outpatient clinic, radiologists, hospital teams as well as to general practitioners. New systems in the UK have incentivised the assessment of bone health in patients suffering hip fracture although the potential for greatest prevention of significant injuries may well lie with supplementation of those patients suffering more minor fragility fractures.

### 4.4 Study Limitations

This is a small study from a single centre and hence it is possible that the results might not be representative of the rates of vitamin D insufficiency in other areas within the UK and indeed in other countries. It does however highlight the need for systems to ensure that patients with fragility fractures receive appropriate preventative medication. One possible confounding factor is that a proportion of patients may have been taking non-prescription supplements which were not included in their hospital admission clerking. This is however unlikely to account for the 81% of patients with a previous fragility fracture who should have been taking supplements but who were not. We do not know how many of the patients not on supplementation may have previously been offered or prescribed them, but either declined or subsequently discontinued for reasons such as side effects. We also do not know the proportion patients prescribed the supplements who actually comply with taking them. In terms of detection of previous fractures, we are limited to seven years of data from our archiving system, and therefore would have missed fractures prior to this or those managed at other institutions.

## CONCLUSION

Vitamin D deficiency is endemic amongst patients suffering hip fractures. Very few patients who had suffered a previous fragility fracture were taking Calcium and Vitamin D supplements when admitted with a hip fracture several years later. This is an opportunity missed.

## Figures and Tables

**Table 1:** Demographics, mobility and level of dependence.

**Factor**	
**Median Age (IQ range)**	85 Years (IQR 79-90)
**Male: Female ratio**	1:3.5
**Median Serum vitamin D level (IQ range)**	32nmol/l (IQR 18-50)
**Pre-admission mobility (n, %)**	
Walked unaided / 1 stick	85, 52.8%
Walked with 2 sticks / frame	19, 11.8%
Wheelchair / bedbound	19, 11.8%
Unknown	27, 16.8%
**Pre-admission level of dependence (n, %)**	
Independent	106, 72.0%
Residential Home	21, 13.1%
Nursing Home	24, 14.9%

**Table 2:** Vitamin D levels and classification.

**Vitamin D Levels**	**Number/Total (%)**
Optimal (>75nmol/l)	14/161 (8.7)
Sufficient (50-75nmol/l)	25/161 (15.5)
Insufficient (25-75nmol/l)	59/161 (36.6)
Deficient (<25nmol/l)	63/161 (39)

**Table 3:** Bone protection being taken amongst patients.

**Bone protection**	**Number/Total (%)**
Calcium & Vitamin D	18/161 (11.4)
Other (alendronic acid & risedronate)	14/161 (8)
None	121/161 (80)

**Table 4:** Patient fragility fracture location.

**Fragility Fracture location**	**Number/Total (%)**
Hip	24/47 (51.1)
Vertebral	9/47 (19.1)
Wrist	6/47 (12.8)
Humerus	4/47 (8.5)
Multiple:	
Hip and Wrist	2/47 (4.2)
Hip and Vertebral	1/47 (2.1)
Vertebral and Wrist	1/47 (2.1)

**Table 5:** Patients with fragility fractures taking Calcium and Vitamin D supplements.

**Previous fragility fracture**	**Number/Total (%)**
Fragility Fracture	47/161 (29)
Receiving Calcium and Vitamin D Supplementation	9/47 (19)
No Calcium and Vitamin D Supplementation	38/47 (81)
